# Supplementary material for: SnoRNAs and miRNAs Networks Underlying COVID-19 Disease Severity
Source: Vaccines (Basel). 2021 Sep 23;9(10):1056. doi: 10.3390/vaccines9101056 (PMC8538251; doi:10.3390/vaccines9101056)
Supplement: Supplementary file 1 [file vaccines-09-01056-s001.zip › vaccines-1347285-supplementary.pdf]

Supplementary Materials for

SnoRNAs and miRNAs networks underlying COVID-19 disease severity

Aijaz Parray[1†], Fayaz Ahmad Mir[2†], Asmma Doudin[3], Ahmad Iskandarani[2], Ibn Mohammed Masud Danjuma[4,5], Rahim Ayadathil Thazhhe Kuni[1], Alaaedin Abdelmajid[6], Ibrahim Abdelhafez[4], Rida Arif[4], Mohammad Mulhim[6], Mohammad Abukhattab[6], Shoukat Rashhid Dar[8], Eyad Elkord[7], Abdul Latif Al-khal[6], Abdel-Naser Elzouki[4,5], Farhan Cyprian[4,9*].

Correspondence to: Farhan.cyprian@gmail.com

This file includes:

Figures S1–S2
Tables S1–S5

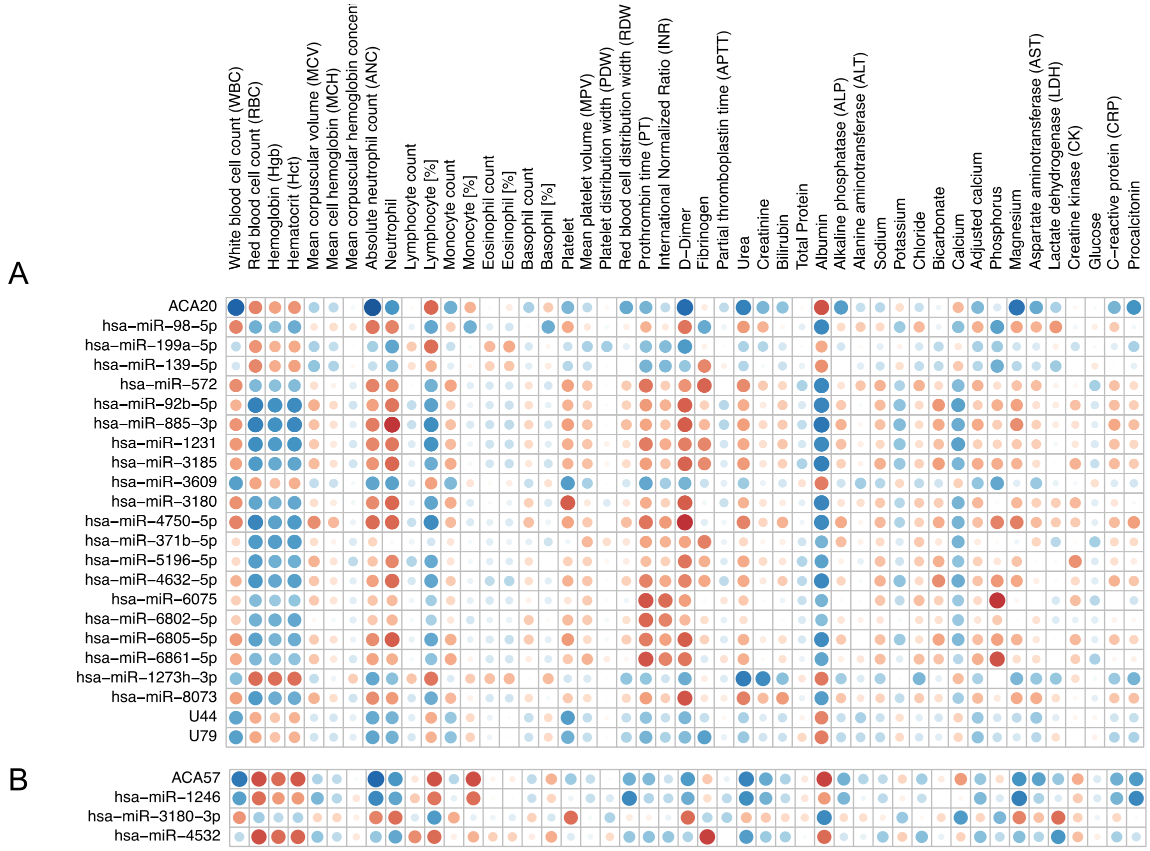

Figure S1. Spearman correlation matrix of clinical markers with uniquely DEMIs and snoRNAs in (A) severe versus mild and (B) severe versus asymptomatic comparisons. Each cell contains a correlation coefficient between the possible pairs of variables, namely Spearman's rho statistic calculated at a significance level of 0.05. Color scale ranges from blue (r = −1) to white (r = 0) to red (r = 1).

(A)

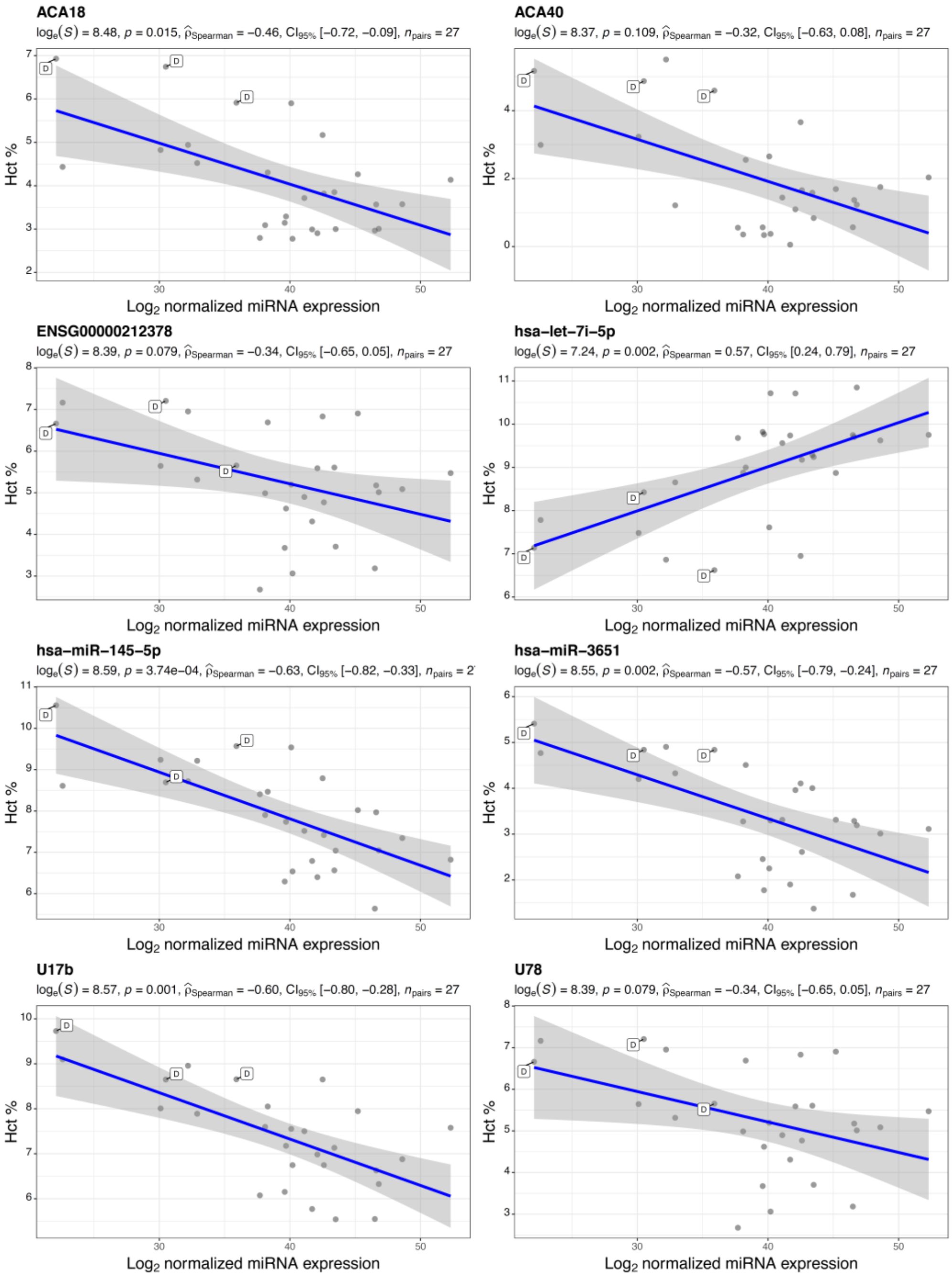
ACA18
$\log_e(S) = 8.48$, $p = 0.015$, $\hat{\rho}_{Spearman} = -0.46$, $CI_{95\%}$ [−0.72, −0.09], $n_{pairs} = 27$
Hct %
Log$_2$ normalized miRNA expression
ACA40
$\log_e(S) = 8.37$, $p = 0.109$, $\hat{\rho}_{Spearman} = -0.32$, $CI_{95\%}$ [−0.63, 0.08], $n_{pairs} = 27$
Hct %
Log$_2$ normalized miRNA expression
ENSG00000212378
$\log_e(S) = 8.39$, $p = 0.079$, $\hat{\rho}_{Spearman} = -0.34$, $CI_{95\%}$ [−0.65, 0.05], $n_{pairs} = 27$
Hct %
Log$_2$ normalized miRNA expression
hsa−let−7i−5p
$\log_e(S) = 7.24$, $p = 0.002$, $\hat{\rho}_{Spearman} = 0.57$, $CI_{95\%}$ [0.24, 0.79], $n_{pairs} = 27$
Hct %
Log$_2$ normalized miRNA expression
hsa−miR−145−5p
$\log_e(S) = 8.59$, $p = 3.74e{-04}$, $\hat{\rho}_{Spearman} = -0.63$, $CI_{95\%}$ [−0.82, −0.33], $n_{pairs} = 2$[illegible]
Hct %
Log$_2$ normalized miRNA expression
hsa−miR−3651
$\log_e(S) = 8.55$, $p = 0.002$, $\hat{\rho}_{Spearman} = -0.57$, $CI_{95\%}$ [−0.79, −0.24], $n_{pairs} = 27$
Hct %
Log$_2$ normalized miRNA expression
U17b
$\log_e(S) = 8.57$, $p = 0.001$, $\hat{\rho}_{Spearman} = -0.60$, $CI_{95\%}$ [−0.80, −0.28], $n_{pairs} = 27$
Hct %
Log$_2$ normalized miRNA expression
U78
$\log_e(S) = 8.39$, $p = 0.079$, $\hat{\rho}_{Spearman} = -0.34$, $CI_{95\%}$ [−0.65, 0.05], $n_{pairs} = 27$
Hct %
Log$_2$ normalized miRNA expression

(B)

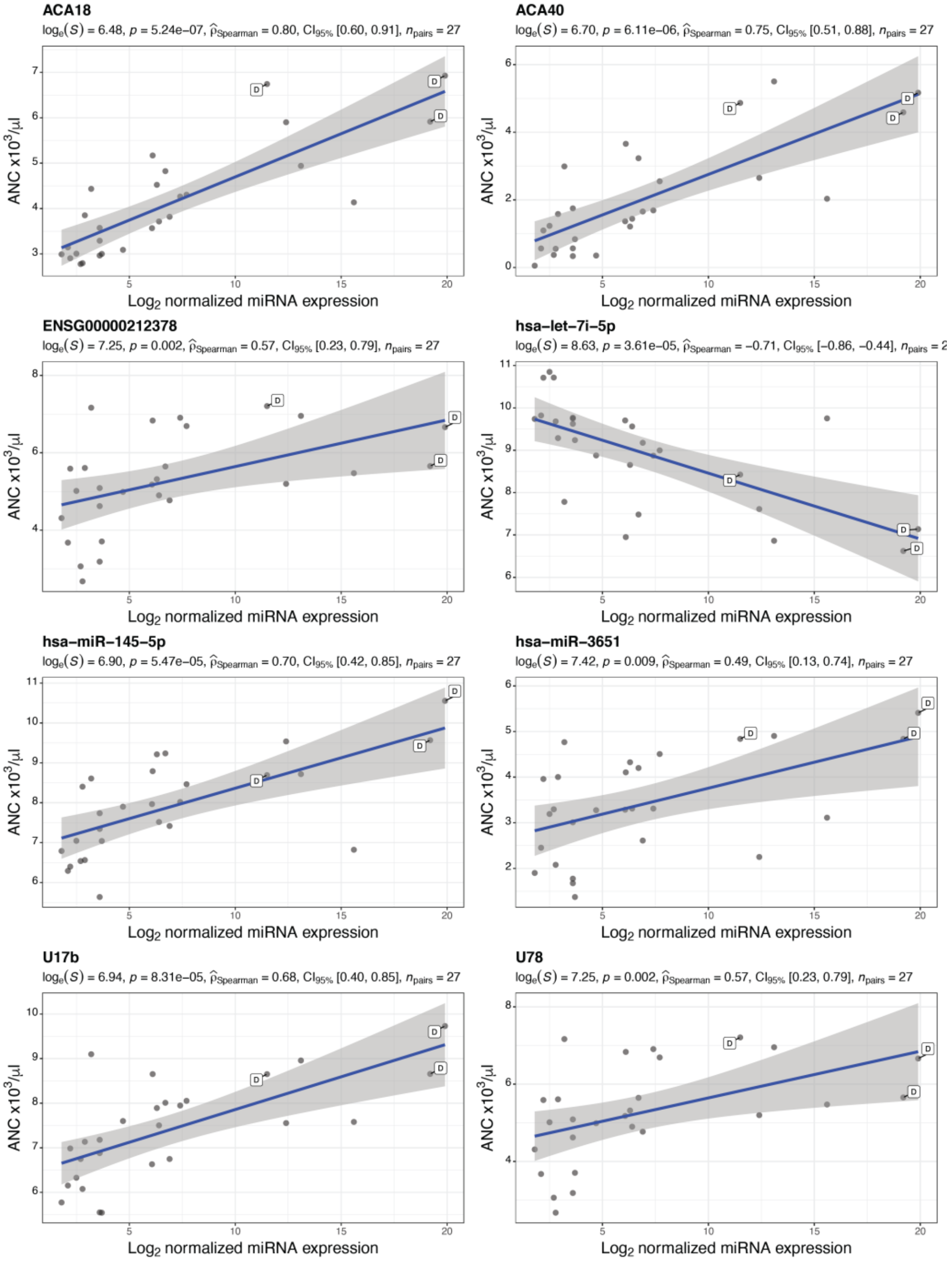

ACA18
$\log_e(S) = 6.48$, $p = 5.24e{-}07$, $\widehat{\rho}_{\text{Spearman}} = 0.80$, $\text{CI}_{95\%}$ [0.60, 0.91], $n_{\text{pairs}} = 27$
ANC x10^3/µl
Log$_2$ normalized miRNA expression
ACA40
$\log_e(S) = 6.70$, $p = 6.11e{-}06$, $\widehat{\rho}_{\text{Spearman}} = 0.75$, $\text{CI}_{95\%}$ [0.51, 0.88], $n_{\text{pairs}} = 27$
ANC x10^3/µl
Log$_2$ normalized miRNA expression
ENSG00000212378
$\log_e(S) = 7.25$, $p = 0.002$, $\widehat{\rho}_{\text{Spearman}} = 0.57$, $\text{CI}_{95\%}$ [0.23, 0.79], $n_{\text{pairs}} = 27$
ANC x10^3/µl
Log$_2$ normalized miRNA expression
hsa-let-7i-5p
$\log_e(S) = 8.63$, $p = 3.61e{-}05$, $\widehat{\rho}_{\text{Spearman}} = -0.71$, $\text{CI}_{95\%}$ [−0.86, −0.44], $n_{\text{pairs}} =$ [illegible]
ANC x10^3/µl
Log$_2$ normalized miRNA expression
hsa-miR-145-5p
$\log_e(S) = 6.90$, $p = 5.47e{-}05$, $\widehat{\rho}_{\text{Spearman}} = 0.70$, $\text{CI}_{95\%}$ [0.42, 0.85], $n_{\text{pairs}} = 27$
ANC x10^3/µl
Log$_2$ normalized miRNA expression
hsa-miR-3651
$\log_e(S) = 7.42$, $p = 0.009$, $\widehat{\rho}_{\text{Spearman}} = 0.49$, $\text{CI}_{95\%}$ [0.13, 0.74], $n_{\text{pairs}} = 27$
ANC x10^3/µl
Log$_2$ normalized miRNA expression
U17b
$\log_e(S) = 6.94$, $p = 8.31e{-}05$, $\widehat{\rho}_{\text{Spearman}} = 0.68$, $\text{CI}_{95\%}$ [0.40, 0.85], $n_{\text{pairs}} = 27$
ANC x10^3/µl
Log$_2$ normalized miRNA expression
U78
$\log_e(S) = 7.25$, $p = 0.002$, $\widehat{\rho}_{\text{Spearman}} = 0.57$, $\text{CI}_{95\%}$ [0.23, 0.79], $n_{\text{pairs}} = 27$
ANC x10^3/µl
Log$_2$ normalized miRNA expression

(C)

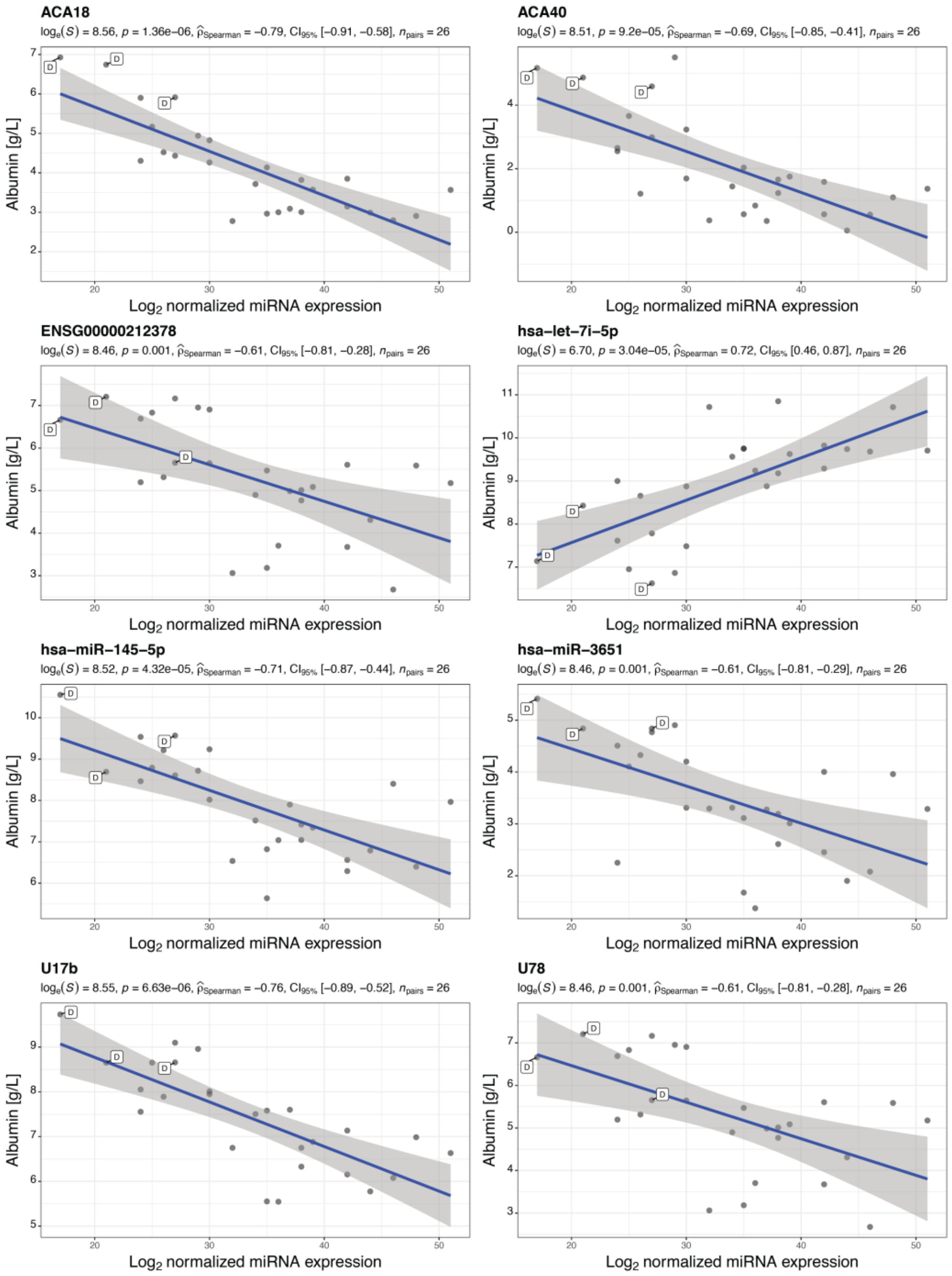

Figure S2. Correlation between expression levels of common differentially expressed miRNAs and snoRNAs (in both severe versus asymptomatic, and severe versus mild comparisons) with (A) hematocrit, (B) absolute neutrophil count (ANC), and (C) albumin. Scatter plots representation with x-axis as Log_2 normalized transcript expression level and y-axis as the clinical variable measurements. Each dot represents a single miRNA/ snoRNAs transcript. Dots that are tagged with D letter represent a deceased patient

measurement. For all statistical tests in the plots, the APA standard for statistical reporting is shown for spearman correlation test including evidence in favor of null over alternative hypothesis, natural logarithm of Bayes Factor, p-value, Spearman's rank correlation coefficient, confidence intervals, and number of observations.

Table S1. Differentially expressed miRNAs and snoRNAs in severe versus asymptomatic and severe versus mild COVID-19 patients. Probe ID and Name, Transcript ID and accession, Log_2 fold change, and FDR values are shown.

Probe ID	Probe Name	Transcript ID	log_2 FC	FDR	Accession
**Severe versus Asymptomatic COVID-19 patients**					
MIMAT0005898_st	20506837	hsa-miR-1246	2.33	0.03	MIMAT0005898
ACA40_x_st	20532669	ACA40	2.15	0.03	ACA40
MIMAT0019071_st	20518933	hsa-miR-4532	2.11	0.03	MIMAT0019071
MIMAT0000437_st	20500755	hsa-miR-145-5p	1.82	0.01	MIMAT0000437
ACA18_x_st	20532628	ACA18	1.75	0.02	ACA18
ACA57_st	20532694	ACA57	1.70	0.02	ACA57
U17b_st	20538140	U17b	1.66	0.02	U17b
ENSG00000212378_s_st	20533073	ENSG00000212378	1.58	0.04	ENSG00000212378
U78_s_st	20538252	U78	1.58	0.04	U78
MIMAT0018071_st	20517902	hsa-miR-3651	1.51	0.03	MIMAT0018071
MIMAT0015058_st	20515610	hsa-miR-3180-3p	-1.55	0.01	MIMAT0015058
MIMAT0000415_st	20500715	hsa-let-7i-5p	-1.60	0.02	MIMAT0000415
**Severe versus Mild COVID-19 patients**					
ACA40_x_st	20532669	ACA40	2.34	0.03	ACA40
MIMAT0017986_st	20517816	hsa-miR-3609	2.26	0.02	MIMAT0017986
ENSG00000212378_s_st	20533073	ENSG00000212378	1.97	0.02	ENSG00000212378
U78_s_st	20538252	U78	1.97	0.02	U78
MIMAT0000231_st	20500399	hsa-miR-199a-5p	1.94	0.05	MIMAT0000231
MIMAT0000250_st	20500432	hsa-miR-139-5p	1.93	0.03	MIMAT0000250
ACA18_x_st	20532628	ACA18	1.87	0.04	ACA18
U79_st	20538254	U79	1.79	0.04	U79
U78_x_st	20538253	U78	1.74	0.04	U78
U17b_st	20538140	U17b	1.69	0.01	U17b
MIMAT0000437_st	20500755	hsa-miR-145-5p	1.69	0.04	MIMAT0000437
MIMAT0018071_st	20517902	hsa-miR-3651	1.61	0.01	MIMAT0018071
ACA20_st	20532631	ACA20	1.59	0.02	ACA20
MIMAT0030416_st	20529133	hsa-miR-1273h-3p	1.56	0.03	MIMAT0030416
U44_st	20538182	U44	1.51	0.05	U44
MIMAT0022977_st	20519405	hsa-miR-4632-5p	-1.53	0.04	MIMAT0022977
MIMAT0027623_st	20525684	hsa-miR-6861-5p	-1.54	0.02	MIMAT0027623

MIMAT0027504_st	20525565	hsa-miR-6802-5p	-1.55	0.02	MIMAT0027504
MIMAT0021128_st	20520577	hsa-miR-5196-5p	-1.57	0.02	MIMAT0021128
MIMAT0004792_st	20504273	hsa-miR-92b-5p	-1.58	0.02	MIMAT0004792
MIMAT0027510_st	20525571	hsa-miR-6805-5p	-1.59	0.04	MIMAT0027510
MIMAT0000096_st	20500179	hsa-miR-98-5p	-1.67	0.04	MIMAT0000096
MIMAT0015065_st	20515617	hsa-miR-3185	-1.72	0.02	MIMAT0015065
MIMAT0003237_st	20504295	hsa-miR-572	-1.79	0.02	MIMAT0003237
MIMAT0019892_st	20519615	hsa-miR-371b-5p	-1.79	0.02	MIMAT0019892
MIMAT0018178_st	20518425	hsa-miR-3180	-1.81	0.02	MIMAT0018178
MIMAT0031000_st	20529783	hsa-miR-8073	-1.82	0.01	MIMAT0031000
MIMAT0019887_st	20519609	hsa-miR-4750-5p	-1.83	0.02	MIMAT0019887
MIMAT0023700_st	20523007	hsa-miR-6075	-1.93	0.03	MIMAT0023700
MIMAT0000415_st	20500715	hsa-let-7i-5p	-1.94	0.02	MIMAT0000415
MIMAT0005586_st	20506779	hsa-miR-1231	-2.00	0.02	MIMAT0005586
MIMAT0004948_st	20505790	hsa-miR-885-3p	-2.03	0.02	MIMAT0004948

Table S2. Mature sequence of annotated differentially expressed miRNAs that are available in miRbase. microRNA names, miRBase accession IDs, and microRNA mature sequences are shown. For hsa-mir-453, reads that map to the annotated mir-4532 locus (many with one mismatch) map exactly to annotated 28S rRNA sequences. The miRNA annotation is therefore likely to be false, and the miRNA was therefore removed from the database.

microRNA name	miRBase accession	microRNA mature sequence
hsa-let-7i-5p	MIMAT0000415	UGAGGUAGUAGUUUGUGCUGUU
hsa-miR-1231	MIMAT0005586	GUGUCUGGGCGGACAGCUGC
hsa-miR-1246	MIMAT0005898	AAUGGAUUUUUGGAGCAGG
hsa-miR-1273h-3p	MIMAT0030416	CUGCAGACUCGACCUCCCAGGC
hsa-miR-139-5p	MIMAT0000250	UCUACAGUGCACGUGUCUCCAGU
hsa-miR-145-5p	MIMAT0000437	GUCCAGUUUUCCCAGGAAUCCCU
hsa-miR-199a-5p	MIMAT0000231	CCCAGUGUUCAGACUACCUGUUC
hsa-miR-3180	MIMAT0018178	UGGGGCGGAGCUUCCGGAG
hsa-miR-3185	MIMAT0015065	AGAAGAAGGCGGUCGGUCUGCGG
hsa-miR-3609	MIMAT0017986	CAAAGUGAUGAGUAAUACUGGCUG
hsa-miR-3651	MIMAT0018071	CAUAGCCCGGUCGCUGGUACAUGA
hsa-miR-371b-5p	MIMAT0019892	ACUCAAAAGAUGGCGGCACUUU
hsa-miR-4632-5p	MIMAT0022977	GAGGGCAGCGUGGGUGUGGCGGA
hsa-miR-4750-5p	MIMAT0019887	CUCGGGCGGAGGUGGUUGAGUG
hsa-miR-5196-5p	MIMAT0021128	AGGGAAGGGGACGAGGGUUGGG
hsa-miR-572	MIMAT0003237	GUCCGCUCGGCGGUGGCCCA
hsa-miR-6075	MIMAT0023700	ACGGCCCAGGCGGCAUUGGUG
hsa-miR-6802-5p	MIMAT0027504	CUAGGUGGGGGGCUUGAAGC
hsa-miR-6805-5p	MIMAT0027510	UAGGGGGCGGCUUGUGGAGUGU
hsa-miR-6861-5p	MIMAT0027623	ACUGGGUAGGUGGGGCUCCAGG
hsa-miR-8073	MIMAT0031000	ACCUGGCAGCAGGGAGCGUCGU
hsa-miR-885-3p	MIMAT0004948	AGGCAGCGGGGUGUAGUGGAUA
hsa-miR-92b-5p	MIMAT0004792	AGGGACGGGACGCGGUGCAGUG
hsa-miR-98-5p	MIMAT0000096	UGAGGUAGUAAGUUGUAUUGUU

Table S3. Sequences of differentially expressed small nucleolar RNAs in severe cases of COVID-19. Transcripts include snoRNA predicted using sequences from RFAM and miRbase. The following snoRNAs transcript IDs are not annotated in miRbase database:

Transcript ID	Sequence
ACA18	GTTGAGGTCTATCCCGATGGGGCTTTTCCTGTAGCCTGCA<br>CATCGTTGGAAACGCCTCATAGAGTAACTCTGTGGTTTTA<br>CTTTACTCACAGGACTATTGTTAGATCTGTGGGAAGGAAT<br>TACAAGACAGTT
ACA20	CTTCCCATTTATTTGCTGCTTGTAGTCTCACAGTGATACGA<br>GCAGTTATACGCATGGGATAAAATAACATTGGGCCACTG<br>TAAATTGAGATGAAGTAACCATTTTCATCTCTTCTGCAGG<br>GACTAGACATTG
ACA40	TGCACTTATGTATGTTTTTGTTTAACGTGGACAAAGACTTA<br>CAGATAGGTGCAAAAAATAAATCCTCTTTTGCAACCCAG<br>AACTCATTGTTCAGTATGAGTTTTGATACATATAAGAAGG<br>GATATTA
ACA57	TTGTCCTGGCCTATTTTTCTGCTCCCCTGTGCTCAGTTCTA<br>ACAGGGTAGTCTGGCAGGACACACAGCAATTCCCTCTCA<br>GTTTAGGAGGGCCGTCCTAAGAATAGGGCTGGCTCTTAA<br>AGGCACGAGAGGACAATT
ENSG00000212378	ATGTAATAATGTTCATCAAATGTCTGACCTGAAATGAGCA<br>TGTAGACAAGTTAATTTAACACTGAAGAA
U17b	TCCAACGTGGATACCCTGGGAGGTCACTCTCCCCAGGCTC<br>TGTCCAAGTGGCATAGGGGAGCTTAGGGCTCTGCCCCAT<br>GATGTACAGTCCCTTTCCACAACGTTGAAGATGAAGCTG<br>GGCCTCGTGTCTGCGCCTGCATATTCCTACAGCTTCCCAG<br>AGTCCTGTGGACAATGACTGGGGAGACAAACCATGCAGG<br>AAACATAT
U44	CCTGGATGATGATAAGCAAATGCTGACTGAACATGAAGG<br>TCTTAATTAGCTCTAACTGACTAA
U78	GTGTAATGATGTTGATCAAATGTCTGACCTGAAATGAGC<br>ATGTAGACAAAGGTAACACTGAAGAA
U79	TACTGTTAGTGATGATTTTAAAATTAAAGCAGATGGGAAT<br>CTCTCTGAGAAAGAAAATGGAGATTAATCTTAAACTGAA<br>ACAGTA

Table S4. Gene Set Enrichment Analysis (GSEA) of differentially expressed miRNAs-targets via Enricher platform and Molecular Signatures Database (MSigDB). Each hallmark gene set is an expressed signature derived by aggregating many MSigDB gene sets to characterize reported biological states or processes.

**Hallmark gene set**	***p*-value**	**q-value**
TNF-alpha Signaling via NF-kB	8.09E-09	1.66E-07
UV Response Dn	6.79E-09	1.66E-07
IL-2/STAT5 Signaling	1.44E-06	1.97E-05
Hypoxia	1.68E-05	1.37E-04
Inflammatory Response	1.68E-05	1.37E-04
Apoptosis	4.89E-05	3.34E-04
PI3K/AKT/mTOR Signaling	6.76E-05	3.96E-04
G2-M Checkpoint	1.62E-04	8.28E-04
Notch Signaling	2.92E-04	1.33E-03
Allograft Rejection	1.32E-03	3.60E-03
Estrogen Response Early	1.32E-03	3.60E-03
Glycolysis	1.32E-03	3.60E-03
Interferon Gamma Response	1.32E-03	3.60E-03
mTORC1 Signaling	1.32E-03	3.60E-03
p53 Pathway	1.32E-03	3.60E-03
IL-6/JAK/STAT3 Signaling	5.31E-03	1.36E-02
Angiogenesis	9.34E-03	1.74E-02
Apical Junction	8.87E-03	1.74E-02
Hedgehog Signaling	9.34E-03	1.74E-02
KRAS Signaling Dn	8.87E-03	1.74E-02
Mitotic Spindle	8.72E-03	1.74E-02
Myogenesis	8.87E-03	1.74E-02
Wnt-beta Catenin Signaling	1.26E-02	2.24E-02
TGF-beta Signaling	2.02E-02	3.46E-02
Myc Targets V2	2.31E-02	3.79E-02

Table. S5. Candidate genes that connect with more than 6 miRNA-target interactions using miRTarBase. *CDKN1A: Cyclin Dependent Kinase Inhibitor 1A, SOCS7: Suppressor Of Cytokine Signaling 7, IGF1R: Insulin Like Growth Factor 1 Receptor, MIDN: Midnolin, LMNB2: Lamin B2.

Gene Symbol*	*p*-value	FDR	Odd ratio	Interactions	microRNAs
CDKN1A	0.00055	0.3	0.318	10	hsa-miR-145-5p, hsa-miR-572, hsa-miR-98-5p, hsa-miR-371b-5p, hsa-let-7i-5p, hsa-miR-6802-5p, hsa-miR-3180, hsa-miR-4632-5p, hsa-miR-6805-5p, hsa-miR-5196-5p
SOCS7	0.00037	0.3	0.206	7	hsa-miR-145-5p, hsa-let-7i-5p, hsa-miR-1273h-3p, hsa-miR-4532, hsa-miR-4632-5p, hsa-miR-92b-5p, hsa-miR-98-5p
IGF1R	0.0252	0.3	0.393	6	hsa-miR-145-5p, hsa-miR-139-5p, hsa-miR-3609, hsa-miR-98-5p, hsa-let-7i-5p, hsa-miR-885-3p
MIDN	0.0148	0.3	0.349	6	hsa-miR-5196-5p, hsa-miR-3609, hsa-miR-4632-5p, hsa-miR-3180, hsa-let-7i-5p, hsa-miR-98-5p
